# Randomized Cross-Over Study of In-Vehicle Cabin Air Filtration, Air Pollution Exposure, and Acute Changes to Heart Rate Variability, Saliva Cortisol, and Cognitive Function

**DOI:** 10.1021/acs.est.2c06556

**Published:** 2023-02-14

**Authors:** Gary Mallach, Robin Shutt, Errol M. Thomson, Frédéric Valcin, Ryan Kulka, Scott Weichenthal

**Affiliations:** † Water and Air Quality Bureau, Health Canada, Ottawa K1A 0K9, Canada; ‡ Environmental Health Science and Research Bureau, Health Canada, Ottawa K1A 0K9, Canada; § Department of Biochemistry, Microbiology and Immunology, Faculty of Medicine, University of Ottawa, Ottawa K1H 8M5, Canada; ∥ Department of Epidemiology, Biostatistics, and Occupational Health, McGill University, Montreal H3A 1G1, Canada

**Keywords:** air pollution, particulate matter, cognition, cortisol, filtration, traffic, intervention

## Abstract

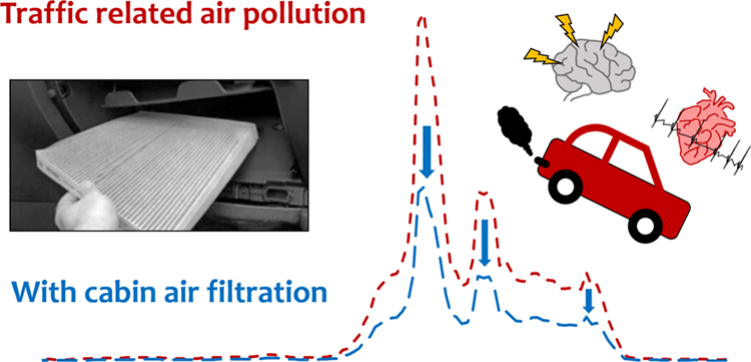

To determine how traffic-related air pollution (TRAP) exposures affect commuter health, and whether cabin air filtration (CAF) can mitigate exposures, we conducted a cross-over study of 48 adults exposed to TRAP during two commutes with and without CAF. Measurements included particulate air pollutants (PM_2.5_, black carbon [BC], ultrafine particles [UFPs]), volatile organic compounds, and nitrogen dioxide. We measured participants’ heart rate variability (HRV), saliva cortisol, and cognitive function. On average, CAF reduced concentrations of UFPs by 26,232 (95%CI: 11,734, 40,730) n/cm^3^, PM_2.5_ by 6 (95%CI: 5, 8) μg/m^3^, and BC by 1348 (95%CI: 1042, 1654) ng/m^3^, or 28, 30, and 32%, respectively. Each IQR increase in PM_2.5_ was associated with a 28% (95%CI: 2, 60) increase in high-frequency power HRV at the end of the commute and a 22% (95%CI: 7, 39) increase 45 min afterward. IQR increases in UFPs were associated with increased saliva cortisol in women during the commute (18% [95%CI: 0, 40]). IQR increases in UFPs were associated with strong switching costs (19% [95%CI: 2, 39]), indicating a reduced capacity for multitasking, and PM_2.5_ was associated with increased reaction latency, indicating slower responses (5% [95%CI: 1, 10]). CAF can reduce particulate exposures by almost a third.

## Abstract 2

Exposure to air pollution while commuting can impact cognitive function, stress hormone levels, and cardiovascular function. Cabin air filtration can help reduce exposure to particulate air pollutants while commuting.

## 1 Introduction

Traffic-related air pollution (TRAP) is linked to adverse cardiopulmonary health and increased mortality, likely through biological mechanisms including systemic inflammation, autonomic dysfunction, and oxidative stress.^1−6^ Recent evidence suggests that longer-term TRAP exposure may impact cognitive function, ranging from mild cognitive impairment to Alzheimer’s disease.^7−9^ The effects of acute exposure to TRAP on cognitive function are less well studied. Previous studies have linked air pollution to physiological stress pathways, including the hypothalamic pituitary axis and autonomic nervous system function.^10,11^

Exposure to the highest levels of TRAP can occur closest to the source, such as in a car in dense traffic.^12,13^ As vehicles emit many pollutants such as NOx, particulate matter (PM), and volatile organic compounds (VOCs), levels of these pollutants along roadways are elevated compared to the urban background. Concentrations within moving vehicles reflect these elevated on-road concentrations, contributing significantly to personal exposures.^14^ For example, a study in Los Angeles found driving time accounted for only 6% of the participants’ days but contributed 33–45% of participants’ ultrafine particle (UFP) exposures.^13^

Reducing in-vehicle pollutant exposures may have sizeable health benefits, and cabin air filtration is a possible, cost-effective exposure mitigation strategy broadly available to the public. Most new vehicles now accommodate a cabin air filter to help reduce in-vehicle TRAP exposures, but their effectiveness in reducing commuter exposures and protecting health remains unclear.

We evaluated the effectiveness of an electrostatic cabin air filter at reducing pollutant exposures and evaluated associations between commuter exposures to air pollutants and pre-clinical changes in physiological endpoints including heart rate variability (HRV), saliva cortisol (a stress hormone), and cognitive function.

## 2 Methods

### 2.1 Study Design

We used a randomized, double-blind, placebo-controlled cross-over design (Figure S1) to evaluate the effectiveness of cabin air filtration at reducing in-vehicle exposures and to determine whether filtration and in-vehicle pollutant concentrations impact acute health endpoints. This randomized design is advantageous in that each subject experiences both exposure conditions at different time points and thus minimizes confounding by individual-level factors that do not vary within subjects over short periods of time (e.g., body mass index). Participants were asked to use the same route and mode of transportation to reach the study site on both study days. On each study day (Monday through Thursday), participants arrived at the central study site in downtown Montreal at 6:30 a.m. for Holter monitor set up and baseline measurements. Participants remained indoors in a dedicated clinical setting for 2 h before the drive, returning to the space after the drive and remaining there until all follow-up measures were completed, within 90 min. Environmental conditions in the space remained clean and stable throughout the study, with indoor air pollution concentrations averaging as follows: <1000/cm^3^ UFPs, 2 μg/m^3^ PM_2.5_, 79 ng/m^3^ Black Carbon, 11 ppb NO_2_, 1.5 μg/m^3^ benzene, and 13.56 μg/m^3^ BTEX. After baseline measurements, study staff drove participants in a minivan for 76 km (approximately 1.5 h, 8:30–10:00 am) along a predefined route and then returned to the central study site (i.e., the commute). The commute consisted of major roads and highways affected by diesel and gasoline vehicles including short segments passing through vehicle tunnels (Figure S2). As noise exposure may impact the health endpoints being evaluated, we took action to maintain a consistent noise level in the vehicles throughout the commutes. This included keeping vehicle windows and doors closed, keeping the ventilation system on a consistent level to generate white noise, playing music from a smooth jazz satellite radio station, and instructing participants and study technicians not to speak during the commute. Furthermore, the cross-over design meant that there were similar traffic conditions across study days, with each participant completing their two commutes on the same day of the week.

#### 2.1.1 Randomization

We randomly assigned participants to travel in one of two minivans on each study day. One vehicle was randomly selected to employ cabin air filtration using an electrostatic air filter while the other had a placebo air filter (i.e., unfiltered air). Following a 1-week washout period participants “crossed over” to the other air supply condition, traveling along the same driving route. Thus, each participant was exposed once to filtered air and once to unfiltered air over two visits. Drivers and study participants were blinded to air supply conditions (filtered or unfiltered) during all commutes. Participants traveled in the same vehicle and sat in the same seat during each trip. Up to three participants rode in the vehicle, and participants were asked to rest quietly and not to engage in discussion during the exposure route. Vehicle ventilation settings were the same for all trips: medium airflow, recirculation off, dashboard vent, and temperature set to 22 °C. Minivans were used to fit multiple participants comfortably and to accommodate the exposure equipment.

#### 2.1.2 Air Filter Selection

Commercially available electrostatic cabin air filters designed to fit Chrysler and Dodge branded minivans were used in this study. These filters were selected based on pilot testing conducted in an Environment and Climate Change Canada laboratory prior to starting the field study, as described in a separate publication.^15^ For this testing, a soot generator emitted particles into a minivan ventilation system to simulate driving conditions. Filter selection was based on the ability of 11 different makes/models of cabin filters to reduce in-cabin concentrations of ultrafine particles (UFP), PM_2.5_, and black carbon. Electrostatic filters were found to be the most effective at removing particulate matter and thus were selected for use in the cross-over study.

### 2.2 Study Population

Study participants (*n* = 48) were mainly students recruited from nearby universities through community advertisements. Most participants lived in downtown Montreal. Prior to enrollment, participants were screened by phone to ensure that they were nonsmokers (in nonsmoking homes), aged between 18 and 65 years, without a chronic disease, and able to provide informed consent. Self-reported mild or moderate asthmatic subjects were included in the study, except those that reported asthma medication use on a regular basis (i.e., daily). Daily inhaled steroidal medications may impact physiological pathways implicated in the effects related to TRAP exposure, such as cortisol production and other stress responses.^16^

Healthy young participants were recruited to minimize impacts of pre-existing cardiac or cognitive conditions on the study results. Our interest was in acute subclinical impacts. Pregnant women, subjects with self-reported respiratory (other than asthma) or cardiovascular illnesses, and people with pacemakers were excluded from the study. Our sample size was limited by the breadth of health measurements collected, and the laboratory’s resources to carry out the measurements. This may have impacted our statistical power. The study was approved by the Health Canada Research Ethics Board and is registered with ClinicalTrials.gov (ID: NCT02277002).

### 2.3 Health Outcomes

#### 2.3.1 Heart Rate Variability

Heart rate variability (HRV) was monitored before, during, and after the commute using an ambulatory ECG (Holter) monitor (GE Seer Light or SEER Light Extend recorders). Measures of HRV were calculated as outlined by the Task Force of the European Society of Cardiology and the North American Society of Pacing and Electrophysiology, and both time (standard deviation of normal-to-normal intervals [SDNN]; root mean square of the successive differences [RMSSD]); and frequency domain measures of HRV (low-frequency spectral power [LF]; high-frequency spectral power [HF]).^17,18^ An expert at the Ottawa Heart Institute conducted all ECG analysis using a GE Healthcare MARS Workstation, using data collected over 5-min intervals preceding the following timepoints: the end of the commute (0 min), 45 min after the commute, and 90 min after the commute.

#### 2.3.2 Saliva Cortisol

Salivary cortisol was measured before, during, and after the commute on each study day (10 min prior to the commute; 45 min into the commute; 10 min postcommute; and 80 min postcommute). The timing of measurements was consistent across days for each participant to control for diurnal patterns. Participants could not eat, drink, or chew gum for 30 min before giving a saliva sample or ingesting a large meal less than 1 h before the saliva sample. Samples were collected using Salivette swabs (Nümbrecht, Germany). Participants rolled the swab in their mouths for 60 s and returned the swab to the Salivette tube. Samples were kept on ice during transport and stored at −20 °C. The saliva was analyzed with a high-sensitivity enzyme-linked immunosorbent assay kit from Salimetrics (State College, PA), and all samples were analyzed in duplicate, with the average of the duplicates used for statistical analyses.

#### 2.3.3 Cognitive Function

The CANTAB testing battery is a cognitive assessment system administered by touch screen. Participants were introduced to the CANTAB tasks during an introductory visit before the study commenced to minimize any learning effects. The CANTAB battery was administered on each study day before and after the commute and included four “tasks” focusing on different cognitive domains and functional areas.

The Attention Switching Task (AST) indicates the costs of multitasking, showing cognitive flexibility, and is indicative of executive function. For this test, an arrow is shown pointing left or right and is displayed on the left or right side of the screen. Participants are asked to indicate either the side of the screen on which the arrow appears or its direction. Sometimes, the arrow’s side and direction are incongruent, whereas sometimes they agree. Longer reaction times for incongruent stimuli are an indication of a Stroop effect (automatic processing being faster than conscious visual control) or *Congruency Cost*. Slower response times for trials in which the rules are changed, versus trials in which the rules are consistent, demonstrate a multitasking effect, or *Switching Cost*. Reaction latencies are measured overall and for congruent and switching trials.^19^

The Reaction Time (RTI) task measures reaction and moving times under simple and 5-choice scenarios. For this task, the subject holds down a button until a spot is shown on the monitor, at which point they move their finger to touch the spot with the same finger. The spot can appear either in a single location (Simple) or in one of five locations (5-choice).^19^

The Rapid Visual Processing (RVP) task measures continuous performance as well as visual sustained attention. For this task, 100 single digits per minute appear in the center of a screen, and the subject must indicate when they see specific number sequences by pressing a button. The outcome measure *A*′ *(A prime)* measures participants’ ability to detect the target sequences, while latency indicates the speed at which participants respond correctly to the target sequence.^19^ This task is associated with the activation of the brain’s frontal and parietal lobes.^20^

The Spatial Working Memory (SWM) task measures subjects’ capacity to retain and process spatial information. For this, subjects search for blue squares by touching larger squares covering them, without revisiting boxes where a blue square was previously uncovered. *Between Errors* measures how often a subject mistakenly returns to a box where a blue square was previously uncovered. The *Strategy* outcome measures whether subjects exhibit a plan to find the tokens, as indicated by whether they start searching from the same box.^19^ SWM can be impaired by damage to the prefrontal cortex and is associated with activations to this area in neuroimaging studies.^21^ Participants completed a practice run of the battery of cognitive functions tests precommute to minimize any training effects that could skew the results and familiarize themselves with the equipment such as buttons and touch screens.

### 2.4 Exposure Assessment

The following air pollutants were simultaneously monitored inside the vehicle and outside the vehicle using instruments mounted on the roof-top: UFPs, PM_2.5_, black carbon, BTEX (sum of benzene, toluene, ethylbenzene, and xylenes), nitrogen dioxide (NO_2_), carbon monoxide (CO), carbon dioxide (CO_2_), temperature, and relative humidity. DustTrak 8520 (TSI, St. Paul, MN) monitors were used to log measurements of PM_2.5_ each minute using a 2.5 μm size-selective inlet and operating at a flow rate of 1.7 liters per minute. Previously conducted in-vehicle and rooftop monitoring using the DustTrak 8520 and collocated gravimetric PM_2.5_ measurements in Canadian cities suggests that the DustTrak 8520 overestimates PM_2.5_ in this environment by a factor of two.^12^ Black carbon was logged at 1 min intervals using an AE51microaetholometer (Magee Scientific, Berkeley, CA). UFP concentrations were measured using the DiSCmini Handheld Nanoparticle Counter (Testo, Lenzkirch, Germany) at 1 min logging intervals. Ninety-minute integrated BTEX concentrations were measured using Summa canisters (6-liter), with subsequent analysis by gas chromatography/mass spectrometry (GC/MS) following the US EPA Method TO-15. Ogawa passive samplers (Ogawa and Company, FL, USA) were used to collect NO_2_ measurements with analysis by ion chromatography. In-vehicle CO_2_ was determined using Vaisala GMW21D monitors (Vaisala Instruments, Helsinki, Finland). Temperature and relative humidity were monitored using HOBO (Onset, Cape Cod, MA, USA) data loggers. In addition, data for ambient temperature, relative humidity, and wind speed were collected from Environment and Climate Change Canada for each study day along with regional estimates of NO_2_ and PM_2.5_. Quality assurance/control was conducted by intercomparing all continuous instruments prior to and postdeployment and deploying blank and duplicate samples for integrated samples. Ogawa NO_2_ samplers were deployed in duplicate, and the median percent precision was 10%. The performance of continuous monitoring equipment was consistent with a previously published method validation study.^22^

### 2.5 Statistical Analysis

Linear regression models were used to estimate the impact of air filter use (as a dichotomous variable) on in-vehicle pollutant concentrations, adjusting for concurrent rooftop concentrations. Linear mixed models with random intercepts for subject were used for all health analyses to account for repeated measurements within subjects; pollutant coefficients were scaled to reflect interquartile range (IQR) increases in pollutant levels (BC = 1818 ng/m^3^, UFP = 40,980/cm^3^, PM_2.5_ = 14 μg/m^3^, and NO_2_ = 21 ppb). We did not adjust for variables that do not meaningfully vary over short periods of time (e.g., age, BMI) or at all (e.g., biological sex) because participants are compared to themselves in the repeated measures cross-over design, and thus, these factors cannot confound the analysis. Only single pollutant models were examined owing to correlations between in-vehicle pollutant concentrations. To estimate associations between filter presence and health outcomes, a dichotomous variable (filter/placebo) was included in the model. In-vehicle temperature was included as a continuous term in all health outcome models along with binary variables indicating participant-reported illness during the previous week. Potential effect modifications by sex were evaluated by including first-order interaction terms as well as through stratified analyses. The impacts of both air filter presence and mean in-vehicle air pollutant concentrations on HRV were examined at three time points, using data collected by the Holter monitor over the 5-min period preceding: the end of the commute (0 min) and 45 and 90 min after the route. Saliva cortisol was evaluated for associations with exposures at three time points; during the commute after 45 min in-vehicle (for this, average PM_2.5_, BC, and UFP exposures were calculated based on preceding 45 min of exposure) and 10 min and 80 min after the commute (using mean in-vehicle concentrations). The saliva cortisol models also included the baseline measurement as a covariate to adjust for daily variability in cortisol levels. All health outcomes were log-transformed prior to analysis to normalize residuals. As a result, the effect estimates represent percent changes in the outcome per IQR increase in pollutant concentration during the commute. All statistical analysis was carried out using STATA 13 (StataCorp LP, College Station, TX, USA).

## 3 Results

### 3.1 Impact of Cabin Air Filter on In-Vehicle Exposures

In-vehicle and rooftop pollutant concentrations were measured during 48 vehicle commutes over 24 weekdays in October and November 2015 (mean duration 94 [SD 10] min) (Tables 1 and S1). Adjusting for concurrent rooftop concentrations, the cabin air filter reduced in-vehicle exposures to UFPs by 26,232 (95%CI: 11,734, 40,730) n/cm^3^, PM_2.5_ by 6 (95%CI: 5, 8) μg/m^3^, and BC by 1348 (95%CI: 1042, 1654) ng/m^3^, or 28, 30, and 32%, respectively. Likewise, the air filter reduced in-vehicle/rooftop ratios of those pollutants by approximately one third, corroborating filtration efficiency results (Table S2). As expected, filtration did not reduce gaseous pollutant concentrations (NO_2_, benzene, total BTEX, or CO). Filter presence did not affect in-vehicle CO_2_ (an indicator of in-vehicle ventilation), indicating that the cabin air filter did not affect air change rates by impeding airflow through the ventilation system.

**Table 1:** Mean In-Vehicle Environmental Conditions with and without a Cabin Air Filter

parameter	avg filtration effectiveness^a^	mean reduction (95%CI)^a^	filter type	*N*	mean	std. dev.	min	max	median *I*/*O* ratio
UFP N/cm^3^	**28%**	**26,232 (11,734, 40,730)**	placebo	23	94,381	47,779	39,088	276,790	0.68
			filter	23	62,606	21,948	32,869	107,863	0.47
PM_2.5_ μg/m^3^	**30%**	**6 (5,8)**	placebo	24	20	9	8	37	0.81
			filter	24	14	7	5	28	0.55
BC ng/m^3^	**32%**	**1348 (1042, 1654)**	placebo	24	4258	2234	1140	9300	0.84
			filter	24	3062	1063	1424	5742	0.59
NO_2_ ppb	–17%	–6 (−20, 9)	placebo	22	35	15	2	79	0.62
			filter	19	40	29	1	114	0.80
Benzene μg/m^3^	2%	0.02 (−0.10, 0.15)	placebo	24	1.62	0.73	0.93	3.25	1.24
			filter	24	1.66	0.60	0.93	2.82	1.33
BTEX μg/m^3^	6%	0.73 (−0.36, 1.82)	placebo	24	13.15	6.99	6.73	31.34	1.41
			filter	24	13.09	5.32	6.96	23.38	1.42
CO ppm	0%	0 (−0.15, 0.14)	placebo	23	1.7	0.3	1.2	2.2	0.92
			filter	24	1.7	0.2	1.3	2.1	1.00
CO_2_ ppm	–2%	–14 (−42, 71)	placebo	24	731	96	648	1117	
			filter	24	745	100	622	1133	
% RH	–3%	–1 (−5, 2)	placebo	24	35	12	19	58	
			filter	24	35	10	18	52	
temperature °C	2%	0.5 (−0.3, 1.4)	placebo	24	22.2	1.7	19.7	26.0	
			filter	24	21.8	1.5	18.1	23.9	

a Adjusted for outdoor-rooftop measurement, except CO_2_. Statistically significant (*p* < 0.05) results are shown in bold.

Regardless of the presence of a cabin air filter, concentrations of UFPs, PM_2.5_, black carbon, CO, and NO_2_ were significantly lower in-vehicle than on the rooftop with indoor/outdoor ratios of these pollutants less than one, indicating that the vehicle affords some protection irrespective of the filter. BTEX (and benzene) concentrations were higher in-vehicles than the rooftop, possibly due to contributions from in-vehicle sources (e.g., self-contamination, interior solvents, or furnishings).

Particulate concentrations increased considerably when passing through vehicle tunnels. Figure S3 shows a typical in-vehicle exposure profile for a driving day, along with the times the vehicle entered the tunnel. Compared to median in-vehicle concentrations, peak in-tunnel concentrations averaged 2.2 times higher for PM_2.5_ and 1.9 times higher for BC and UFPs (see the shaded areas in Figure S3).

### 3.2 Study Population

Mean BMI and baseline systolic and diastolic blood pressure measurements in our participants (53% female; mean age 26 (range 29–49) years) are indicative of a population in good health (Table S3). Continuous rhythm disturbances precluded seven participant days of HRV measurement data from four participants. One participant was excluded from analysis for medical reasons unrelated to the study. One participant underwent the same treatment condition twice and was excluded from the analysis of health outcomes by the filter status but was included in the health outcome models incorporating pollutant concentrations.

### 3.3 Heart Rate Variability

**Figure 1:**
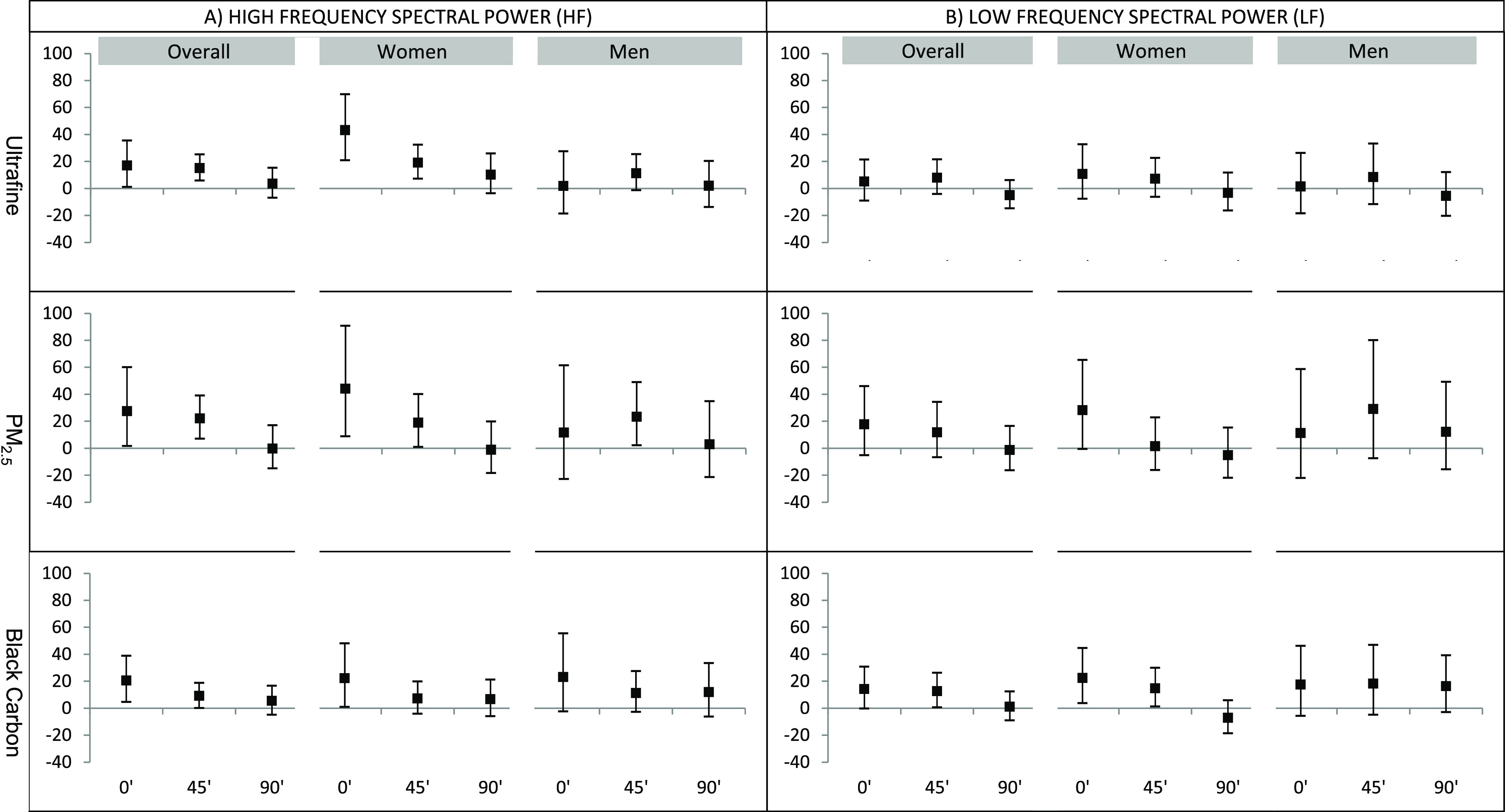
Percent change in frequency domain HRV per IQR change in pollutant at the end of the ride, 45′ post, and 90′ postexposure. Adjusted for sickness reported in the past week and indoor temperature. Percent changes in HRV are per IQR change in pollutant: UFP = 40,980/cm^3^, PM_2.5_ = 14 μg/m^3^, BC = 1818 ng/m^3^.

**Figure 2:**
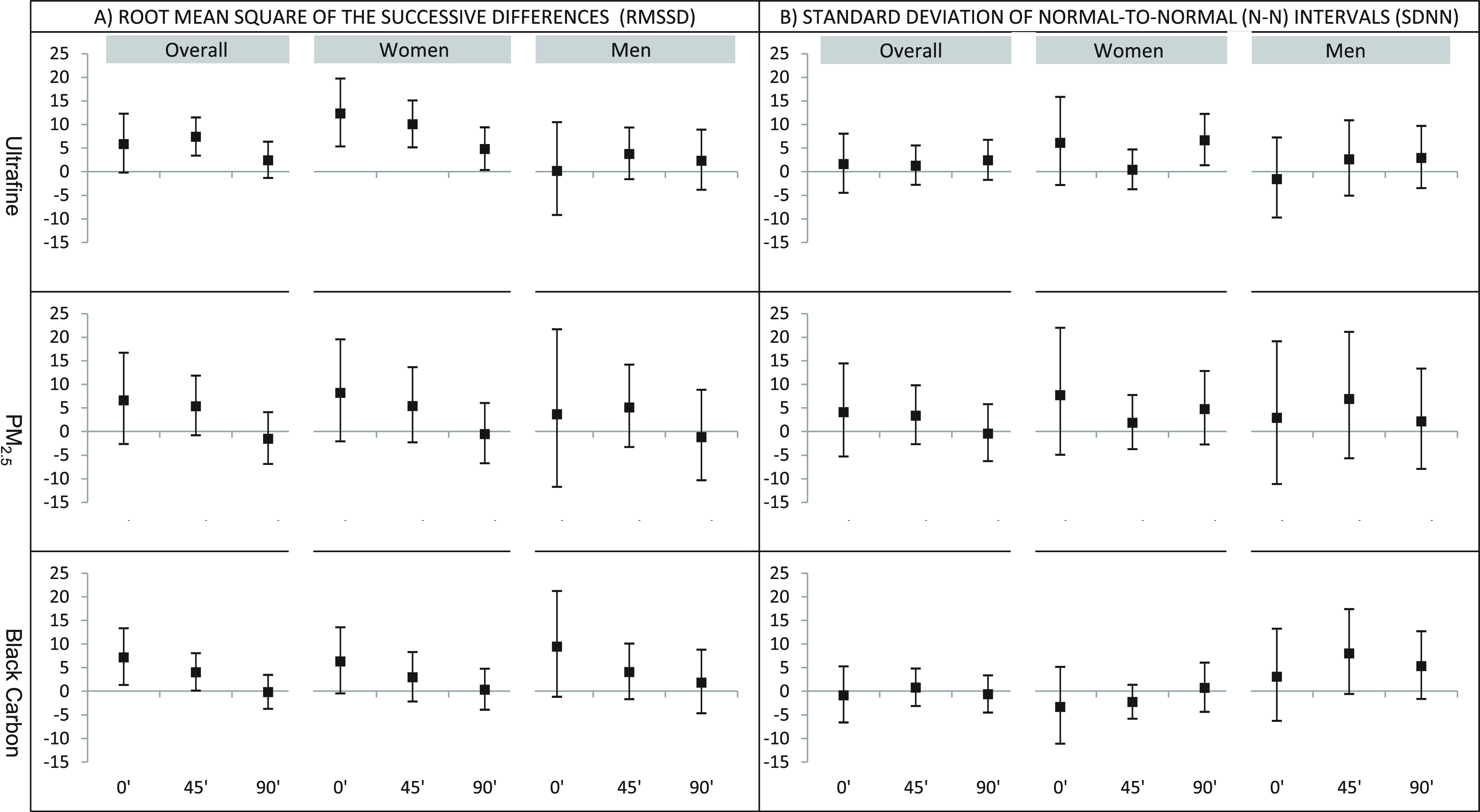
Percent change in time domain HRV per IQR increase in pollutant at the end of the ride, 45′ post, and 90′ post exposure. Adjusted for sickness reported in the past week and indoor temperature. Percent changes in HRV are per IQR change in pollutant: UFP = 40,980/cm^3^, PM_2.5_ = 14 μg/m^3^, BC = 1818 ng/m^3^.

Figures 1 and 2 illustrate percent changes in HRV associated with IQR increases in mean in-vehicle concentrations of UFPs, PM_2.5_, and BC (along with results for NO_2_, in Tables S4 and S5) Associations between frequency domain HRV (HF and LF) and IQR increases in in-vehicle pollutant exposures are shown in Figure 1 (Table S4). Mean in-vehicle exposure to UFPs, PM_2.5_, and BC was each associated with increased HF at the end of the drive (Figure 1a). Diminished effects can be seen 45 min postexposure, and, by 90 min, no substantial increases persist. Similarly, BC is associated with increased LF at the end of the drive and 45 min post.

Effects were generally more pronounced in women than in men. For women, in-vehicle exposure to UFPs, PM_2.5_, BC, and NO_2_ were each associated with increased HF at the end of the drive and up to 45 min afterward; men only displayed substantial effects for PM_2.5_, at 45 min postcommute. BC was associated with increased LF at the end of the drive and 45 min post in women (Figure 1b and Table S4). The interaction term for sex was significant for HF responses to UFPs at the end of the commute (0 min) (*p* < 0.05).

Associations between time domain HRV (RMSSD and SDNN) and IQR increases in in-vehicle pollutant exposures are shown in Figure 2 (Table S5). Overall, UFPs were associated with increases in RMSSD 45 min after the commute, and black carbon was associated with increases in RMSSD immediately after the commute and 45 min later. Generally, higher in-vehicle pollutant exposures were associated with increased RMSSD, although effects subsided over 90 min after the commute. There were sex differences in RMSSD responses to UFPs at 0- and 45-min postcommute (*p* < 0.05), with larger effects seen in women than in men. In-vehicle NO_2_ exposure was associated with increased HF and RMSSD in women, up to 45 min postcommute (Tables S4 and S5). In men, NO_2_ was associated with decreased LF at 0 min postcommute, and the interaction term for sex indicated that this association differed significantly by sex (*p* < 0.01). Air filter use was generally associated with reduced HRV (Table S6). Stronger associations were seen at the 60 min mark and end of the drive.

### 3.4 Saliva Cortisol

After 45 min in-vehicle, IQR increases in UFPs associated with 18% increases to cortisol levels in women, whereas in men, PM_2.5_, BC, and BTEX were associated with 38, 30, and 28% decreases, respectively (Table 2). Ten minutes after the commute, IQR increases in UFPs were associated with decreased cortisol in men. The presence of the cabin air filter was not associated with changes to cortisol. During the commute, sex differences in cortisol responses were statistically significant for PM_2.5_ (*p* < 0.005), BC (*p* < 0.005), and BTEX (*p* < 0.05), with pollutant exposures generally being associated with increased cortisol levels in women and decreased cortisol in men.

**Table 2:** Percent Change in Saliva Cortisol per IQR Increase in Pollutant or Filter Presence

saliva collection time		PM_2.5_	UFP	BC	BTEX	NO_2_	filter+
after 45 min in vehicle^a^	overall	–11 (−26, 8)	2 (−14, 20)	–7 (−19, 7)	–8 (−22, 9)	2 (−11, 17)	2 (−17, 26)
	women	12 (−8, 36)	**18 (0, 40)**	4 (−9, 20)	5 (−11, 25)	1 (−15, 20)	–5 (−23, 18)
	men	**–38 (−55, −15)**	–19 (−39, 7)	**–30 (−46, −10)**	**–28 (−46, −3)**	–1 (−19, 21)	7 (−30, 62)
10 min after commute	overall	–6 (−22, 14)	–9 (−21, 5)	3 (−10, 19)	1 (−13, 19)	–6 (−17, 7)	5 (−14, 29)
	women	0 (−21, 26)	–3 (−17, 14)	12 (−5, 33)	10 (−10, 33)	–2 (−18, 17)	3 (−22, 35)
	men	–15 (−35, 13)	**–25 (−43, −1)**	–7 (−26, 16)	–7 (−27, 17)	–9 (−23, 7)	8 (−17, 41)
80 min after commute	overall	–3 (−18, 15)	1 (−11, 15)	0 (−11, 14)	1 (−12, 17)	9 (−3, 22)	2 (−14, 21)
	women	–5 (−22, 17)	2 (−12, 17)	–2 (−15, 14)	5 (−12, 24)	**18 (2, 38)**	15 (−8, 45)
	men	4 (−20, 36)	–6 (−28, 23)	8 (−13, 33)	1 (−19, 26)	5 (−10, 23)	–6 (−26, 21)

a In-vehicle exposures preceding saliva collection used for PM2.5, UFP, and BC analyses.

Adjusted for baseline cortisol measurement, sickness reported in the past week, and indoor temperature. Percent changes in saliva cortisol are per IQR change in pollutant: PM_2.5_ = 14 μg/m^3^, UFP = 40,980/cm^3^, BC = 1818 ng/m^3^, BTEX = 8.2 ppm, NO_2_ = 21 ppb. Filter+ indicates percent change when the filter is present versus when it is not (placebo). 95% confidence intervals shown in parentheses.

### 3.5 Cognitive Function

In-vehicle exposures were associated with decrements to neurocognitive function, most frequently for BC (Table 3). On the attention switching task, IQR increases in particulate pollutants were associated with decreases in congruency cost, meaning that increased exposure was associated with a greater deficit in trials focused on automatic processing than those involving conscious visual control. Importantly, UFP exposure was associated with strong switching costs, indicating reduced capacity for multitasking. Particulate pollutants and BTEX were associated with increases in reaction latency, indicating slower responses for all test modes. On the reaction time test, particulate exposures were associated with small increases to simple and 5-choice reaction times, while NO_2_ was associated with slower movement times.

**Table 3:** Percentage Change in Neurocognitive Outcome Score per IQR Increase in Pollutants or by Filter Presence^a^

neurocognitive outcome	pollutant/filter status					
	PM_2.5_	BC	UFP	BTEX	NO_2_	filter (+)
AST: congruency cost	–12 (−32, 14)	**–22 (−34, −8)**	–1 (−17, 19)	–12 (−29, 10)	–7 (−21, 10)	–3 (−29, 31)
AST: switching cost	17 (−7, 47)	9 (−6, 27)	**19 (2, 39)**	13 (−7, 38)	1 (−14, 20)	–7 (−28, 20)
AST: reaction latency	**5 (1, 10)**	**5 (2, 8)**	**3 (0, 7)**	**5 (1, 9)**	1 (−3, 4)	–3 (−8, 2)
AST: reaction latency congruent	**5 (0, 10)**	**5 (2, 8)**	**3 (0, 7)**	**6 (2, 10)**	1 (−3, 5)	–3 (−8, 2)
AST: reaction latency switching	**7 (2, 13)**	**6 (3, 10)**	**4 (1, 8)**	**9 (4, 13)**	1 (−3, 6)	–3 (−8, 3)
RTI: simple reaction time	3 (−1, 8)	2 (−1, 5)	2 (−2, 5)	1 (−3, 6)	1 (−2, 4)	0 (−5, 5)
RTI: simple movement time	–2 (−6, 2)	**–3 (−6, 0)**	–3 (−5, 1)	2 (−2, 5)	**5 (1, 8)**	3 (−1, 8)
RTI: 5-choice reaction time	2 (−1, 6)	**2 (0, 5)**	2 (0, 5)	0 (−3, 3)	1 (−2, 3)	–1 (−4, 3)
RTI: 5-choice movement time	–3 (−6, 1)	**–4 (−6, −1)**	–1 (−4, 2)	0 (−3, 4)	**5 (2, 9)**	2 (−2, 7)
RVP: *A*′ prime	0 (−1, 1)	**–1 (−2, 0)**	0 (−1, 1)	–1 (−2, 0)	0 (−1, 1)	–1 (−2, 0)
RVP: latency	–2 (−6, 2)	–1 (−4, 1)	–2 (−5, 1)	0 (−3, 4)	1 (−2, 4)	**6 (1, 10)**
SWM: between errors	8 (−19, 42)	**22 (1, 47)**	11 (−10, 35)	10 (−14, 40)	12 (−8, 37)	–14 (−36, 14)
SWM: strategy	**4 (0, 8)**	2 (0, 4)	1 (−1, 4)	1 (−2, 4)	–1 (−4, 2)	–3 (−7, 0)

a AST = attention switching task; RTI = reaction time; RVP = rapid visual processing; SWM = spatial working memory. Adjusted for sickness reported in the past week and indoor temperature. Statistically significant results in bold (*p* < 0.05). Percent changes in neurocognitive outcome scores are per IQR change in pollutant: PM_2.5_ = 14 μg/m^3^, BC = 1818 ng/m^3^, UFP = 40,980/cm^3^, BTEX = 8.2 ppm, NO_2_ = 21 ppb. Filter (+) indicates percent change when the filter is present versus when it is not (placebo). 95% confidence intervals shown in parentheses.

On the rapid visual processing task, BC was associated with small decreases in A’ prime, indicating lower sensitivity to the target sequences. Similarly, spatial working memory was diminished, as evidenced by increased *Between Errors* and lower scores for *Strategy*.

In general, filter presence itself was not associated with changes to neurocognitive outcomes. We did not examine sex-related differences in cognitive function outcomes due to ethical considerations.

## 4 Discussion

### 4.1 Exposure

Although Canadians and Americans only spend an average of five to six percent of their time in vehicles,^23,24^ this time contributes substantially to total daily air pollution exposure.^12,13^ In this study, we found that cabin air filtration can reduce in-vehicle particulate exposures by approximately one third, compared to placebo. This is similar to findings from other studies that included measurements of the efficiency of cabin air filters.^25^ As expected, the filter only removed particulates as there is no mode of action for electrostatic filters to alter gaseous pollutants. However, activated charcoal filters have been shown to reduce BTEX.^15^ TRAP concentrations were notably elevated in tunnels, as was found in Hong Kong,^26^ and vehicle rooftop PM_2.5_ concentrations were substantially higher than ambient measurements at the regional regulatory monitoring station, reflecting the close proximity to mobile source TRAP emissions. Average rooftop PM_2.5_ and BC concentrations were similar to those found previously in Montreal,^12^ and generally lower than exposures in Europe,^27^ likely due to the higher prevalence of diesel vehicles on European roadways.

Because of considerable daily variability in TRAP concentrations during the study, filter presence itself was not a perfect indicator of in-vehicle pollutant exposure; indeed, some participants were exposed to higher in-vehicle pollutant concentrations during the filtered exposure because of substantially higher outdoor pollutant concentrations on the “filtered” study day. As a result, the in-vehicle pollutant measurements were more appropriate for use in health analyses, owing to less exposure misclassification.

While this study only looked at the benefit of cabin air filtration when using “outdoor air” mode, using “recirculation” mode can also reduce in-vehicle exposures to pollutants from outside the vehicle, particularly when a cabin air filter is in place. However, using the recirculation mode for extended periods of time may lead to elevated concentrations of other contaminants, including CO_2_ from vehicle passengers, which may impact cognitive function.^28^

In-vehicle TRAP exposures may affect commuters’ cardiovascular and stress responses and affect cognitive function. We demonstrated the effectiveness of cabin air filtration at reducing in-vehicle pollution exposures. Cabin air filtration using regularly maintained, best-available technologies may reduce in-vehicle exposures and protect health.

### 4.2 Heart Rate Variability

Heart rate variability is used to evaluate the health and balance of the autonomic nervous system, with both increased and decreased HRV identified as risk factors for cardiac mortality.^29^ Few studies have examined whether modifying a vehicle’s ventilation system can decrease exposure to air pollution and in turn affect HRV. In a study of 60 participants, cabin air conditioning with a filter in place was able to reduce PM_2.5_ by more than half, with associated increases to standard deviation of normal-to-normal intervals (SDNN) and root mean square of successive differences (RMSSD).^30^ Another cross-over study examining the effect of in-vehicle exposure to TRAP of HRV did not find any significant differences between filtered and unfiltered rides, although it did note general post-exposure changes from baseline measurements.^31^ Elevated levels of TRAP were also shown to affect HRV in a study of 11 taxi drivers during the Beijing Olympic Games.^32^ In our study, exposure to TRAP while commuting was associated with changes in some measures of HRV. In-vehicle exposure to PM_2.5_ and BC affected HRV within a very short time frame, and effects persisted at least 45 min after the commute. Two measures of HRV, HF power, and the RMSSD appeared to change with TRAP exposure. Both of these measures are associated with changes in the parasympathetic nervous system input into the heart, suggesting a shift toward parasympathetic nervous system activation.^33^ In general, effects of air pollution exposure on HRV were more pronounced in women; this is consistent with research, suggesting that women already have a higher ratio of parasympathetic control of cardiac function.^34^ The finding of a sex difference is the effects of TRAP, which is also consistent with certain studies finding women more susceptible to air pollution than men, including cardiovascular mortality due to PM_2.5_ exposure.^35,36^

### 4.3 Saliva Cortisol

Saliva cortisol is a noninvasive indicator of active free cortisol, a glucocorticoid used as a biomarker of stress, and HPA axis activation.^37,38^ Air pollutants may initiate a neuroendocrine stress response, stimulating the hypothalamic–pituitary–adrenal (HPA) axis and altering cortisol levels in the body; over time, this may cause disease.^10^ Research linking exposure to traffic-related air pollutants—notably NO_2_ and particulate matter—and HPA axis activation (e.g., cortisol levels) has produced divergent results, possibly owing to differences in study design (i.e., exposure-response time intervals analyzed), emission sources, and the populations examined. NO_2_ exposures averaged over the preceding year were associated with a flattened diurnal slope in adolescents, indicating abnormal cortisol response,^39^ and with a higher awakening cortisol level in a cohort of 45–85-year-old adults.^40^ Exposure to PM2.5 averaged over the preceding 4–10 months was associated with decreased morning cortisol levels among overweight and obese youth.^41^ In a cross-over trial of healthy students, 9-day exposure to elevated PM2.5 was associated with higher morning cortisol, compared to when exposed to filtered air.^42^ Controlled exposure for 2 h to diluted diesel exhaust acutely increased the plasma cortisol level in a randomized double-blind cross-over study,^43^ providing causal support for a relationship between exposure to TRAP and cortisol response. In experiments with rats, the rapid increase of the glucocorticoid corticosterone following pollutant exposure was linked to effects throughout the body, including the modulation of biological pathways in the brain, highlighting the systemic consequences of pollutant effects on the HPA axis.^44^

Our study found that common TRAP pollutant exposures were associated with marked changes to saliva cortisol, indicating that commuter exposures may impact acute stress responses, particularly during the period of exposure, with effects subsiding shortly after the exposure is reduced. In the present study, cortisol increased in women in relation to some TRAP constituents, while men showed a significant decline for several other TRAP constituents. Given the time of the commute (morning), we would expect to see either constant or declining cortisol levels in all participants across the period of the commute, due to the decline following the cortisol awakening response (CAR),^45^ although some of the differences in responses may also be attributable to sex differences in the CAR. Glucocorticoids, in the form of cortisol in humans, help regulate cardiovascular, metabolic, immunologic, developmental, reproductive, and homeostatic processes. By the way of the central nervous system, they also help regulate memory, cognition, mood, and sleep. Although the temporary activation of the HPA axis is a normal stress response, chronic or recurrent activation can dysregulate the HPA axis, which may impact metabolic, neurobehavioral, cardiovascular, and reproductive processes.^46,47^ Together with evidence that air pollution exposure stimulates the HPA axis, the similarity in disease pathways and outcomes associated with both air pollution and HPA axis dysfunction may implicate HPA axis dysfunction as a cause of some of the negative health effects of air pollution.^10^

### 4.4 Cognitive Function

Air pollution exposure may reduce cognitive function, with associated effects ranging from mild cognitive impairment to Alzheimer’s disease and dementia.^7−9^ The mechanisms underlying air pollution’s effect on the brain remain uncertain, although likely involve a variety of pathways including neuroinflammation, dysregulation of the HPA axis, oxidative stress, and even the translocation of inhaled particles through the circulatory system into the brain itself.^48^ Notably, Chen et al. showed that those living near major roadways had a higher incidence of dementia, particularly for those living in major cities.^49^ Non-dementia cognitive function is also linked with exposure to PM_2.5_ and other air contaminants.^9,50,51^ Few studies have examined the impacts of short-term (e.g., hourly) particulate air pollution exposure on cognitive function, and most have relied on central site air pollution monitoring for exposure data rather than using personal or indoor measurements. One study of 90 young adults found that 24 h personal PM_2.5_ exposures measured 3 days prior were associated with reduced Stroop test results and decreased executive function.^52^ Another study of healthy adults exposed participants to low, ambient air pollution, and PM_2.5_ from candle burning or air pollution exposure from 30 min of commuting (walking, cycling, bus, or train) next to a major road. Both of these exposures similarly reduced cognitive function as per the Mini-Mental State Examination (MMSE), indicating that particulate matter exposure was likely the causative agent. The commuter exposure also impacted detection speed in a selective attention test.^53^ Likewise, in a population of 10,228 Lumosity (brain-training game) users, exposure to PM_2.5_ on the day of gameplay was associated with declined performance on a selective attention task.^54^ Finally, in a study of 302 office workers across multiple urban settings, indoor PM_2.5_, particularly above 12 μg/m^3^, was associated with slower reaction times and reduced accuracy on Stroop and addition–subtraction cognitive function assessments.^55^ While the impacts of air pollution on cognitive function remain understudied, these studies (and our own) indicate that air pollution exposure may impact driving ability and postcommute job or educational performance. We found in-vehicle TRAP exposures diminished key areas of cognitive function shortly after exposure including attention switching, a measure of executive function and cognitive flexibility, and reaction time. This may have particular relevance to public safety as PM_2.5_ has been associated with increased traffic accidents.^56,57^

### 4.5 Strengths and Limitations

Strengths of this study include the use of a double-blind cross-over design where neither the study participants nor the study staff (apart from the lead investigator responsible for installing filters in the vehicles) were aware of which vehicle had the cabin air filter in place on a given day. As a cross-over study, participants acted as their own control in the two filter conditions, reducing variability and confounding from time in-varying factors. As with all interventional studies in humans, the sample size was limited by what was feasible, reducing the power of the study, although this was offset by the statistically efficient cross-over design. We did not adjust for multiple comparisons in our analysis but focused on the size and direction of the effect, along with the 95% confidence intervals. This may have increased the type I error rate.

## Supporting Information Available

Cross-over study design; driving route in Montreal, QC; in-vehicle, rooftop, and ambient environmental conditions during filtered and unfiltered periods; effect of air filtration on indoor–outdoor ratios of measured air pollutants and descriptive statistics; time series of in-vehicle PM2.5, black carbon, and UFPs for a typical daily drive; population characteristics; percent change in frequency domain HRV per IQR change in pollutant at the end of the ride, 45′ post, and 90′ postexposure; percent change in time domain HRV per IQR change in pollutants at the end of the ride, 45′ post, and 90′ post exposure per IQR change in in-vehicle pollutant exposure; percent changes in HRV associated with the cabin air filter; baseline scores on the CANTAB neurocognitive test battery; and schedule of participant activities during the day of study visit (PDF)
