# Supplementary material for: Randomized Cross-Over Study of In-Vehicle Cabin Air Filtration, Air Pollution Exposure, and Acute Changes to Heart Rate Variability, Saliva Cortisol, and Cognitive Function
Source: Environ Sci Technol. 2023 Feb 14;57(8):3238–47. doi: 10.1021/acs.est.2c06556 (PMC9979657; doi:10.1021/acs.est.2c06556)
Supplement: Supplementary file 1 — es2c06556_si_001.pdf [file es2c06556_si_001.pdf]

Supplemental Information: A Randomized Crossover Study of In-Vehicle Cabin Air Filtration, Air Pollution Exposure, and Acute Changes to Heart Rate Variability, Saliva Cortisol and Cognitive Function

Gary Mallach,[1]* Robin Shutt,[2] Errol M. Thomson,[3,4] Frédéric Valcin,[1] Ryan Kulka,[1] Scott Weichenthal[1,5]

[1] Water and Air Quality Bureau, Health Canada, Ottawa, Canada

[2] Population Studies Division, Environmental Health Science and Research Bureau, Health Canada, Ottawa, Canada

[3] Hazard Identification Division, Environmental Health Science and Research Bureau, Health Canada, Ottawa, Canada

[4] Department of Biochemistry, Microbiology and Immunology, Faculty of Medicine, University of Ottawa, Ottawa, Canada

[5] McGill University, Department of Epidemiology, Biostatistics, and Occupational Health

*Corresponding Author:

Gary Mallach

Water and Air Quality Bureau

Health Canada

Ottawa, Canada

Email: gary.mallach@hc-sc.gc.ca

## Contents

Figure S1: Crossover Study Design, showing participants randomized into two groups and undergoing a series of two treatments (filter and placebo) with one week washout period between visits........................................ 3

Supplemental Figure S2: Driving Route in Montreal, QC. ........................................................................... 4

Supplemental Table S1: In-vehicle, Rooftop, and Ambient environmental conditions during filtered and unfiltered periods. .......................................................................................................................... 5

Supplementary Table S2: Effect of air filtration on Indoor-Outdoor ratios of measured air pollutants, and descriptive statistics ......................................................................................................................... 6

Supplemental Figure S3: Time series of in-vehicle $PM_{2.5}$, Black Carbon, and UFPs for a typical daily drive, showing tunnel related peaks in grey (Pont-tunnel Louis-Hippolyte-La Fontaine tunnel Ville-Marie).................. 7

Table S3: Population characteristics.......................................................................................................... 8

Supplemental Table S4: Percent change in Frequency Domain HRV per IQR change in pollutant at end of ride, 45’ post, and 90’ post exposure .................................................................................................... 9

Supplemental Table S5: Percent change in Time Domain HRV per IQR change in pollutant at end of ride, 45’ post, and 90’ post exposure per IQR change in in-vehicle pollutant exposure.................................................. 10

Supplemental Table S6: Percent changes in HRV associated with Cabin Air filter (95% Confidence Interval)..... 11

Table S7: Baseline scores on CANTAB neurocognitive test battery prior to commute........................................ 12

Table S8: Schedule of Participant activities during day of study visit. ............................................................... 13

Figure S1: Crossover Study Design, showing participants randomized into two groups and undergoing a series of two treatments (filter and placebo) with one week washout period between visits.

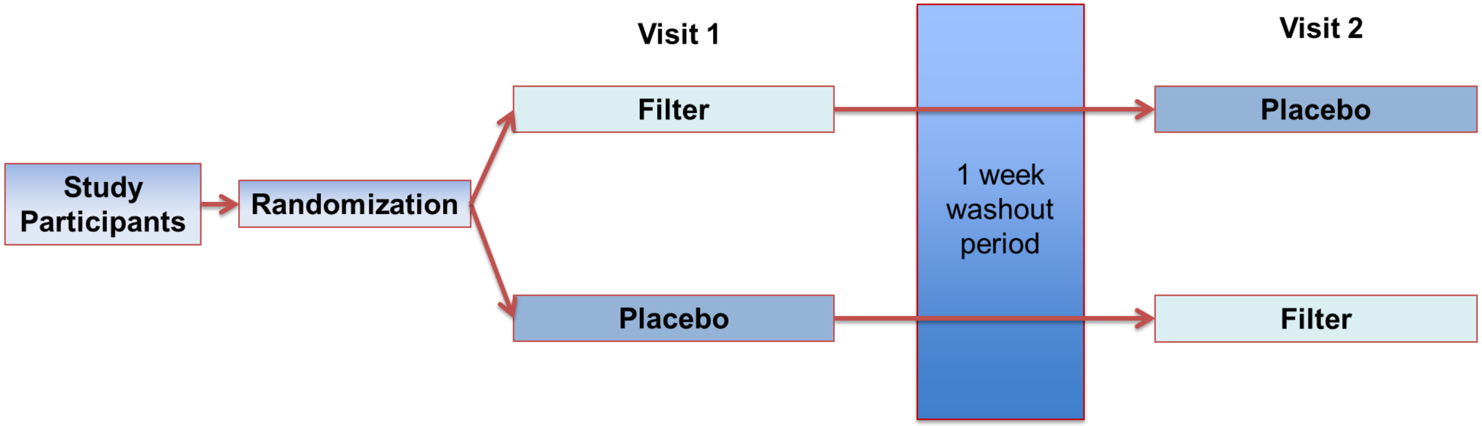

Supplemental Figure S2: Driving Route in Montreal, QC.

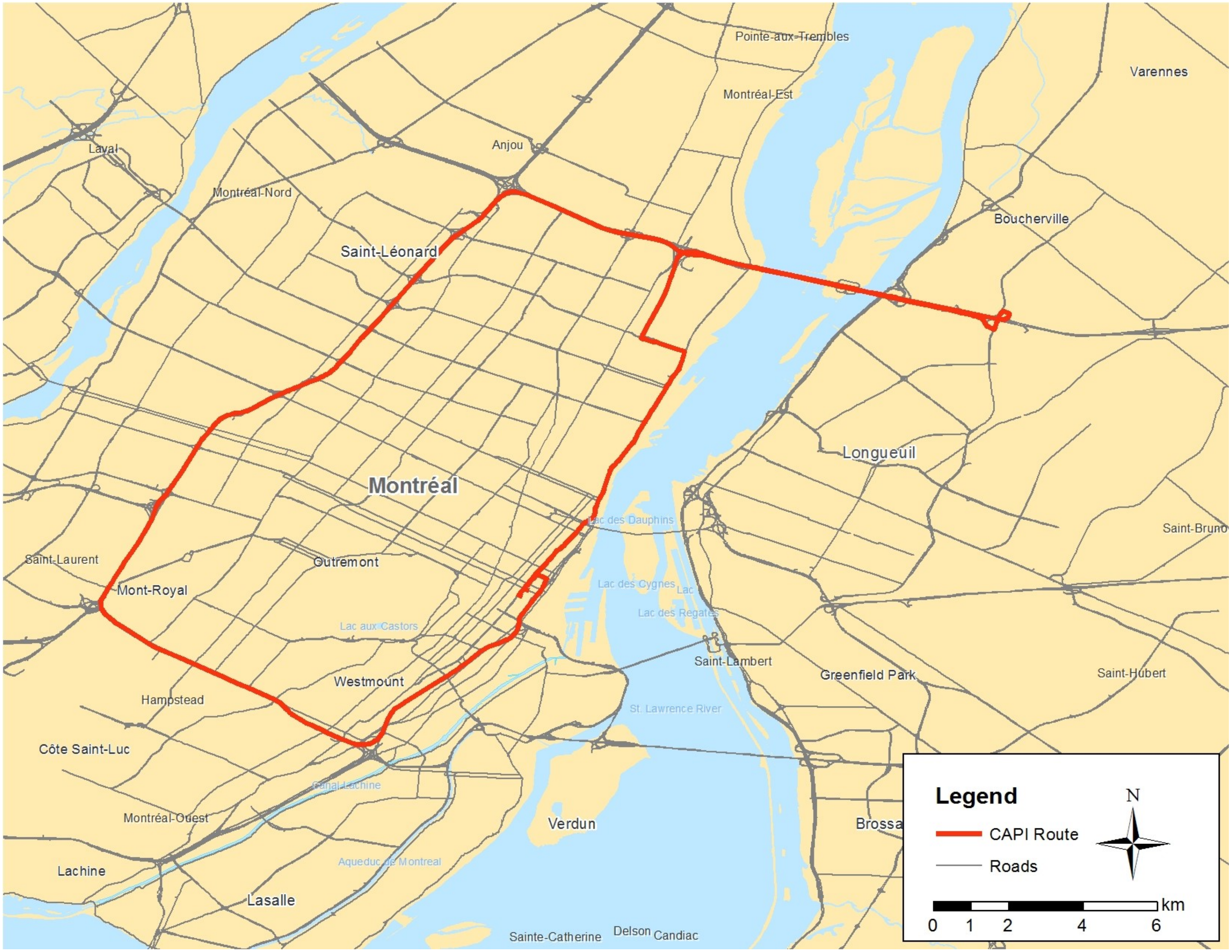

Supplemental Table S1: In-vehicle, Rooftop, and Ambient environmental conditions during filtered and unfiltered periods.

| | | Without Cabin Air Filter | | | | | | With Cabin Air Filter | | | | | |
|---|---|---|---|---|---|---|---|---|---|---|---|---|---|
| *Parameter* | *Location* | *N* | *Mean* | *Median* | *Std. Dev.* | *Min* | *Max* | *N* | *Mean* | *Median* | *Std. Dev.* | *Min* | *Max* |
| $PM_{2.5}$ ug/m3 | In-vehicle | 24 | 20 | 18 | 9 | 8 | 37 | 24 | 14 | 13 | 7 | 5 | 28 |
| | Rooftop | 24 | 25 | 21 | 13 | 10 | 56 | 24 | 25 | 23 | 13 | 10 | 59 |
| | NAPS* | 24 | 10 | 10 | 4.82 | 1 | 19 | 24 | 10 | 10 | 4.82 | 1 | 19 |
| $NO_2$ ppb | In-vehicle | 22 | 35 | 36 | 15 | 2 | 79 | 19 | 40 | 31 | 29 | 1 | 114 |
| | Rooftop | 21 | 65 | 49 | 56 | 1 | 279 | 23 | 55 | 45 | 37 | 1 | 142 |
| | NAPS* | 24 | 14 | 14 | 6 | 5 | 28 | 24 | 13.5 | 13.5 | 6.3 | 5 | 28 |
| BC ng/m3 | In-vehicle | 24 | 4,258 | 3,731 | 2,234 | 1,140 | 9,300 | 24 | 3,062 | 3,047 | 1,063 | 1,424 | 5,742 |
| | Rooftop | 23 | 5,002 | 4,564 | 2,325 | 1,860 | 11,047 | 24 | 5,158 | 5,165 | 1,679 | 2,554 | 8,655 |
| UFP N/cm3 | In-vehicle | 23 | 94,381 | 86,882 | 47,779 | 39,088 | 276,790 | 23 | 62,606 | 57,423 | 21,948 | 32,869 | 107,863 |
| | Rooftop | 24 | 144,462 | 140,759 | 68,746 | 50,356 | 370,173 | 23 | 134,892 | 124,380 | 49,799 | 60,464 | 242,091 |
| Benzene ug/m3 | In-vehicle | 24 | 1.62 | 1.26 | 0.73 | 0.93 | 3.25 | 24 | 1.66 | 1.49 | 0.60 | 0.93 | 2.82 |
| | Rooftop | 24 | 1.25 | 1.01 | 0.51 | 0.70 | 2.23 | 24 | 1.30 | 1.11 | 0.52 | 0.74 | 2.44 |
| BTEX ug/m3 | In-vehicle | 24 | 13.15 | 10.60 | 6.99 | 6.73 | 31.34 | 24 | 13.09 | 12.04 | 5.32 | 6.96 | 23.38 |
| | Rooftop | 24 | 9.19 | 7.52 | 5.07 | 4.09 | 22.10 | 24 | 9.77 | 8.56 | 5.02 | 4.03 | 20.49 |
| CO ppm | In-vehicle | 23 | 1.7 | 1.6 | 0.3 | 1.2 | 2.2 | 24 | 1.7 | 1.6 | 0.2 | 1.3 | 2.1 |
| | Rooftop | 23 | 2.0 | 1.7 | 0.9 | 1.1 | 4.5 | 24 | 1.8 | 1.6 | 0.7 | 1.0 | 3.5 |
| $CO_2$ ppm | In-vehicle | 24 | 731 | 717 | 96 | 648 | 1117 | 24 | 745 | 737 | 100 | 622 | 1133 |
| % RH | In-vehicle | 24 | 35 | 32 | 12 | 19 | 58 | 24 | 35 | 35 | 10 | 18 | 52 |
| | Rooftop | 24 | 46 | 45 | 12 | 26 | 67 | 24 | 44 | 42 | 12 | 26 | 67 |
| Temperature°C | In-vehicle | 24 | 22.2 | 22.3 | 1.7 | 19.7 | 26.0 | 24 | 21.8 | 21.7 | 1.5 | 18.1 | 23.9 |
| | Rooftop | 24 | 12.8 | 12.8 | 5.4 | 2.2 | 23.3 | 24 | 13.3 | 14.4 | 5.0 | 3.8 | 22.6 |

* National Air Pollutant Surveillance: Ambient measurement from Canadian regulatory monitor

Supplementary Table S2: Effect of air filtration on Indoor-Outdoor ratios of measured air pollutants, and descriptive statistics

| *Parameter* | *% Reduction* | *Reduction(95%CI)* | | *Median* | Mean | Std. Dev. |
|---|---|---|---|---|---|---|
| UFP N/cm$^3$ | **-31%** | **-0.21 (-0.35, -0.07 )** | Placebo | 0.68 | 0.69 | 0.30 |
| | | | Filter | 0.47 | 0.48 | 0.12 |
| $PM_{2.5}$ ug/m$^3$ | **-32%** | **-0.26 (-0.30, -0.21 )** | Placebo | 0.81 | 0.81 | 0.10 |
| | | | Filter | 0.55 | 0.56 | 0.07 |
| BC ng/m$^3$ | **-29%** | **-0.24 (-0.29, -0.18** ) | Placebo | 0.84 | 0.83 | 0.11 |
| | | | Filter | 0.59 | 0.59 | 0.06 |
| $NO_2$ ppb | -80% | -3.12 (-9.44, 3.20 ) | Placebo | 0.62 | 3.92 | 13.59 |
| | | | Filter | 0.8 | 0.80 | 0.36 |
| Benzene ug/m3 | 0% | 0.00 (-0.09, 0.10 ) | Placebo | 1.24 | 1.30 | 0.18 |
| | | | Filter | 1.33 | 1.30 | 0.15 |
| BTEX ug/m3 | -3% | -0.04 (-0.17, 0.09 ) | Placebo | 1.41 | 1.46 | 0.21 |
| | | | Filter | 1.42 | 1.42 | 0.23 |
| CO ppm | 5% | 0.05 (-0.14, 0.23 ) | Placebo | 0.92 | 0.98 | 0.32 |
| | | | Filter | 1 | 1.02 | 0.31 |

Statistically significant ($p<0.05$) results are shown in bold.

Supplemental Figure S3: Time series of in-vehicle $PM_{2.5}$, Black Carbon, and UFPs for a typical daily drive, showing tunnel related peaks in grey (Pont-tunnel Louis-Hippolyte-La Fontaine tunnel Ville-Marie)

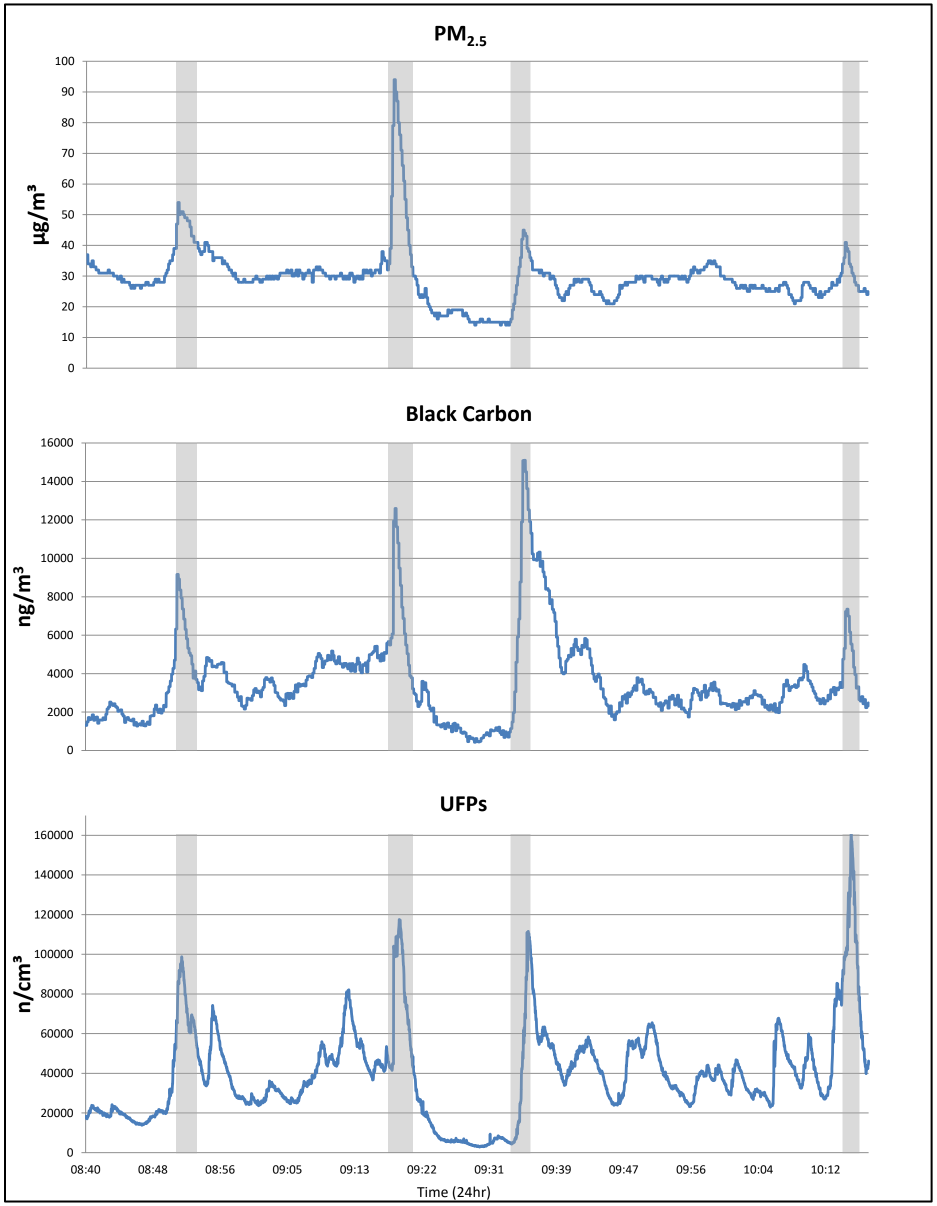

Table S3: Population characteristics

| Population Characteristic | N | Mean ± S.D. (Range) |
|---|---|---|
| Sex: Male / Female | 48 | 23 / 25 |
| Age (years) | 48 | 26 ± 8 (18 - 58) |
| BMI (kg/m$^2$) | 48 | 23 ± 4 (18 - 33) |
| Systolic BP (mmHg)* | 93 | 106 ± 10 (85 - 132) |
| Diastolic BP(mmHg)* | 93 | 69 ± 9 (42 - 97) |
| LF (msec$^2$)* | 85 | 1900 ± 1090 (300 - 5819) |
| HF (msec$^2$)* | 85 | 743 ± 606 (72 - 3513) |
| rMSSD (msec)* | 85 | 107 ± 29 (50 - 195) |
| SDNN (msec)* | 85 | 44 ± 17 ( 18 - 107) |

*Measured before the commute

Supplemental Table S4: Percent change in Frequency Domain HRV per IQR change in pollutant at end of ride, 45’ post, and 90’ post exposure

| | HF | | | LF | | |
|---|---|---|---|---|---|---|
| | 0’ | 45’ | 90’ | 0’ | 45’ | 90’ |
| UFP | | | | | | |
| Overall | **17(1 to 35)** | **15(6 to 25)** | 3.5(-7 to 15) | 5(-9 to 21) | 8(-4 to 22) | -5(-15 to 6) |
| Men | 2(-19 to 28) | 11(-1 to 25) | 1.9(-14 to 20) | 1(-19 to 26) | 8(-12 to 33) | -6(-20 to 12) |
| Women | **43(21 to 70)** | **19(7 to 32)** | 10(-4 to 26) | 11(-8 to 33) | 7(-6 to 23) | -3(-16 to 12) |
| $PM_{2.5}$ | | | | | | |
| Overall | **28(2 to 60)** | **22(7 to 39)** | -0(-15 to 17) | 18(-5 to 46) | 12(-7 to 34) | -1(-16 to 16) |
| Men | 12(-23 to 61) | **23(2 to 49)** | 2.8(-22 to 35) | 11(-22 to 59) | 29(-7 to 80) | 12(-16 to 49) |
| Women | **44(9 to 91)** | **19(1 to 40)** | -1(-19 to 20) | 28(-1 to 65) | 1(-16 to 23) | -5(-22 to 15) |
| BC | | | | | | |
| Overall | **20(5 to 39)** | 9(0 to 19) | 5.4(-5 to 17) | **14(0 to 31)** | **13(1 to 26)** | 1(-9 to 12) |
| Men | 23(-2 to 55) | 11(-3 to 27) | 12(-6 to 33) | 17(-6 to 46) | 18(-5 to 47) | 16(-3 to 39) |
| Women | **22(1 to 48)** | 7(-4 to 20) | 6.8(-6 to 21) | **22(4 to 45)** | **15(1 to 30)** | -7(-19 to 6) |
| $NO_2$ | | | | | | |
| Overall | 8(-7 to 25) | 8(-1 to 17) | 3.3(-7 to 14) | -6(-19 to 9) | -5(-15 to 7) | 0(-9 to 11) |
| Men | -3(-21 to 20) | 4(-6 to 15) | 5.4(-8 to 21) | **-21(-35 to -5)** | -9(-26 to 11) | -6(-18 to 9) |
| Women | **26(2 to 54)** | **14(1 to 28)** | -1(-13 to 14) | 13(-6 to 36) | 0(-13 to 15) | 12(-4 to 30) |

Adjusted for sickness reported in past week, and indoor temperature. Statistically significant results in bold ($p<0.05$). Percent changes in HRV are per IQR change in pollutant: UFP= 40,980/$cm^3$, $PM_{2.5}$=14 μg/$m^3$, BC=1818 ng/$m^3$, $NO_2$=21 ppb.

Supplemental Table S5: Percent change in Time Domain HRV per IQR change in pollutant at end of ride, 45' post, and 90' post exposure per IQR change in in-vehicle pollutant exposure

| | RMSSD | | | SDNN | | |
|---|---|---|---|---|---|---|
| | 0' | 45' | 90' | 0' | 45' | 90' |
| UFP | | | | | | |
| Overall | 6(0 to 12) | **7(3 to 11)** | 2(-1 to 6) | 2(-4 to 8) | 1(-3 to 6) | 2(-2 to 7) |
| Men | 0(-9 to 10) | 4(-2 to 9) | 2(-4 to 9) | -2(-10 to 7) | 3(-5 to 11) | 3(-4 to 10) |
| Women | **12(5 to 20)** | **10(5 to 15)** | **5(0 to 9)** | 6(-3 to 16) | 0(-4 to 5) | **7(1 to 12)** |
| $PM_{2.5}$ | | | | | | |
| Overall | 7(-3 to 17) | 5(-1 to 12) | -2(-7 to 4) | 4(-5 to 14) | 3(-3 to 10) | 0(-6 to 6) |
| Men | 4(-12 to 22) | 5(-3 to 14) | -1(-10 to 9) | 3(-11 to 19) | 7(-6 to 21) | 2(-8 to 13) |
| Women | 8(-2 to 19) | 5(-2 to 14) | -1(-7 to 6) | 8(-5 to 22) | 2(-4 to 8) | 5(-3 to 13) |
| BC | | | | | | |
| Overall | **7(1 to 13)** | **4(0 to 8)** | 0(-4 to 3) | -1(-7 to 5) | 1(-3 to 5) | -1(-5 to 3) |
| Men | 9(-1 to 21) | 4(-2 to 10) | 2(-5 to 9) | 3(-6 to 13) | 8(-1 to 17) | 5(-2 to 13) |
| Women | 6(-1 to 13) | 3(-2 to 8) | 0(-4 to 5) | -3(-11 to 5) | -2(-6 to 1) | 1(-4 to 6) |
| NO2 | | | | | | |
| Overall | 2(-4 to 8) | **4(0 to 9)** | 1(-3 to 5) | 0(-5 to 7) | -1(-4 to 2) | -2(-6 to 3) |
| Men | -3(-11 to 7) | 2(-4 to 9) | 2(-4 to 9) | -5(-12 to 3) | 0(-4 to 5) | 1(-4 to 8) |
| Women | **7(0 to 15)** | **7(2 to 13)** | 2(-3 to 7) | 9(0 to 18) | -2(-6 to 2) | 0(-6 to 6) |

Adjusted for sickness reported in past week, and indoor temperature. Statistically significant results in bold ($p<0.05$).
Percent changes in HRV are per IQR change in pollutant: UFP= 40,980/$cm^3$, $PM_{2.5}$=14 μg/$m^3$, BC=1818 ng/$m^3$, $NO_2$=21 ppb.

Supplemental Table S6: Percent changes in HRV associated with Cabin Air filter (95% Confidence Interval)

| Parameter | Group | In vehicle 30 mins | In-vehicle 60 mins | 0’ (End of Drive) | 45' Post | 90' Post |
|---|---|---|---|---|---|---|
| LF | Overall | -5(-23, 16) | **-27(-43, -6)** | -16(-35, 9) | -10(-28, 12) | 7(-11, 27) |
| | Men | -18(-39, 11) | **-36(-55, -8)** | -28(-49, 4) | -30(-53, 4) | 5(-23, 43) |
| | Women | 5(-21, 39) | -19(-40, 9) | -5(-33, 34) | 11(-10, 37) | 8(-13, 33) |
| HF | Overall | -12(-31, 13) | -19(-36, 2) | **-25(-42, -4**) | -8(-19, 5) | 15(-3, 35) |
| | Men | -9(-30, 20) | -18(-40, 10) | -24(-49, 11) | -11(-27, 7) | 19(-10, 57) |
| | Women | -12(-40, 29) | -17(-41, 15) | -24(-46, 6) | -2(-17, 16) | 9(-11, 34) |
| SDNN | Overall | 0(-10, 11) | -9(-20, 5) | -8(-18, 3) | -2(-9, 4) | -1(-6, 6) |
| | Men | 6(-5, 18) | -3(-23, 22) | -8(-22, 10) | -6(-18, 7) | -2(-12, 9) |
| | Women | -2(-15, 13) | -14(-27, 1) | -8(-21, 7) | 0(-6, 7) | -2(-9, 6) |
| RMSSD | Overall | -8(-16, 0) | -6(-15, 3) | **-10(-19, -1)** | -5(-10, 1) | 2(-4, 8) |
| | Men | -6(-17, 5) | -9(-19, 3) | **-16(-28, -1)** | -4(-11, 5) | 0(-10, 11) |
| | Women | -9(-20, 4) | -3(-15, 11) | -4(-14, 8) | -4(-10, 2) | 3(-4, 9) |

Adjusted for sickness reported in past week, and indoor temperature. Statistically significant results in bold ($p<0.05$). Results indicate the percent change when the filter is present, versus when it is not (placebo).

Table S7: Baseline scores on CANTAB neurocognitive test battery prior to commute.

| Variable | Obs (n) | Mean | Std.Dev | Min | Max |
|---|---|---|---|---|---|
| AST: Congruency cost (ms) | 92 | 63 | 39 | -23 | 146 |
| AST: switching cost (ms) | 94 | 137 | 96 | -24 | 417 |
| AST: Reaction latency (ms) | 94 | 551 | 116 | 366 | 872 |
| AST: Reaction latency congruent (ms) | 94 | 523 | 111 | 342 | 793 |
| AST: Reaction Latency switching (ms) | 94 | 631 | 155 | 373 | 1019 |
| RTI: Simple reaction Time (ms) | 93 | 330 | 70 | 228 | 631 |
| RTI: Simple movement time (ms) | 93 | 575 | 141 | 305 | 969 |
| RTI: 5-choice reaction time (ms) | 93 | 344 | 61 | 248 | 537 |
| RTI: 5-choice movement time (ms) | 93 | 568 | 138 | 306 | 975 |
| RVP: A' prime (% of sequences detected) | 94 | 94 | 5 | 80 | 100 |
| RVP: Latency (ms) | 94 | 356 | 55 | 247 | 633 |
| SWM: Between errors (# incorrect) | 94 | 12 | 15 | 0 | 65 |
| SWM: Strategy (# of strategic trials) | 94 | 28 | 7 | 19 | 44 |

Table S8: Schedule of Participant activities during day of study visit.

| **Time** | **Participant Location** | **Participant data collected** | **Comment** |
|---|---|---|---|
| 07:00-08:30 | Laboratory | Questionnaire;<br>Cognitive function practice test;<br>Saliva cortisol *baseline* measurement (*10- minutes before commute*); | Participants arrived in building lobby at 06:30. |
| 08:30-10:00 | In-vehicle | Heart rate variability (*end of commute*);<br>Saliva cortisol *(after 45 minutes in-vehicle)* | Air pollutant data collected on vehicle rooftop and within cabin while participants were in-vehicle. |
| 10:00-12:00 | Laboratory | Saliva cortisol *(10- and 80-minutes post-commute);*<br>Heart rate variability *(assessed 45- and 90-minutes post-commute);*<br>Cognitive function testing *(50 minutes post-commute)* | Participants departed after providing all necessary health measurements. Participants each had 2 study visits, 1 week apart (cabin air filter and placebo). |
